# A new emergency during the latest phase of the COVID-19 pandemic: access to healthcare services by patients with non-communicable diseases

**DOI:** 10.3389/fmed.2023.1261063

**Published:** 2023-10-12

**Authors:** Caterina De Sarro, Rosa Papadopoli, Maria Carmela Morgante, Claudia Pileggi

**Affiliations:** ^1^Department of Health Sciences, University of Catanzaro "Magna Græcia", Catanzaro, Italy; ^2^FAS@UMG Research Center, Department of Health Science, School of Medicine, University of Catanzaro, Catanzaro, Italy

**Keywords:** COVID-19 pandemics, frailty, health services accessibility, non-communicable, disease

## Abstract

**Background:**

The aim of our study was to investigate the impact of the COVID-19 pandemic on the healthcare and the disease management of patients affected by non-communicable diseases (NCDs), by exploring, specifically, the obstacles encountered in the access to healthcare services during the latest phase of the pandemic.

**Methods:**

This cross-sectional study was carried out among subjects attending the anti-SARS-CoV2 vaccination clinic in a Teaching Hospital of Southern Italy. To be included in the study, subjects had to be affected by at least one NCD, such as diabetes, hypertension, respiratory and heart diseases, renal and liver chronic conditions, immunodeficiency disorders due to cancer, or being kidney or liver transplant recipients.

**Results:**

Among the 553 subjects who completed the questionnaire, the 39.4% (95% IC = 35.3–43.6) experienced obstacles in the access to healthcare services in the six months prior to the enrollment. The most frequent canceled/postponed healthcare services were the visits for routine checks for NCDs (60.6, 95% IC = 53.9–67), control visits of more complex diseases as cancer or transplantation (17.3, 95% IC = 12.6–22.8), and scheduled surgery (11.5, 95% IC = 7.7–16.4). The patients who experienced canceled/postponed healthcare services were significantly more likely to suffer from 3 or more NCDs (*p* = 0.042), to be diabetics (*p* = 0.038), to have immunodeficiency disorders (*p* = 0.028) and to have consulted GP at least once (*p* = 0.004).

**Conclusion:**

Our results appear to be fundamental for guiding the choices of providers in order to concentrate organizational efforts to recover and reschedule missed appointments, where applicable, of the most fragile patients by virtue of age and chronic conditions.

## 1. Introduction

The sudden emergence of SARS-COV2, the cause of a pandemic that has led to more than 750 million confirmed cases and nearly 7 million deaths globally (1), has put a strain on the healthcare systems of countries around the world. In Italy, the law enforcement action was based on a complete lockdown during the first wave of the pandemic (March–June 2020) (2), followed by a series of more or less restrictive measures depending on the epidemiological trend of the infection, which characterized the second (November 2020–March 2021) and the third (March–April 2021) phase of the COVID-19 pandemic, up to the fourth phase characterized by a progressive easing of the restrictive measures which, starting from the end of April 2021 (3, 4), led to the end of the state of emergency in March 2022 (5).

To deal with the emergency, the reallocation of resources was required to strengthen the healthcare activities considered to be essential compared to the non-essential ones (6). Several studies have been performed to evaluate the remodulation of the access to healthcare services during the early phases of the pandemic, specifically since the start of the pandemic through December 2020. These studies have shown that, in 2020, there has been a relevant reduction of outpatient healthcare (7), primary care visits (8, 9), preventive interventions such as screening and vaccinations (7, 10, 11) and elective procedures (7, 12, 13) compared with the preceding years, and in most cases the comparison was with 2019. Instead, very poor evidence is available on the healthcare services utilization during the latest phase of the pandemic, characterized by a more sustainable management of the COVID-19, mostly attributable to the effectiveness of vaccinations, which completely changed the course of SARS-CoV2 infection in all categories of vaccinated subjects, including the frailest (14–16).

According to WHO’s 2020 Global Health Estimates (17), non-communicable diseases (NCDs) affect more people each year and are responsible for more than 70% of all deaths worldwide. The burden of NCDs will continue to grow over the coming decades, mainly due to cardiovascular diseases, with deaths rising by more than 2 million since 2000 to nearly 9 million in 2019, and to neoplastic diseases (18) with, in 2018, 18 million people in the world affected by cancer and more than 9 million deaths. Also, deaths from diabetes globally have more than double between 2000 and 2019 and account for the NCDs with the largest percentage increase of all WHO regions.

The management of NCDs, due to their chronic nature, often require repeated and prolonged interactions with the healthcare system in order to have access to pharmacological, surgical, rehabilitative therapies and regular monitoring, essential to determine if the treatment plan is on track and the patient is achieving results. Not getting the care needed often has devastating consequences for people living with NCDs. A recent meta-analysis (19) highlighted that the risk of death across the seven major cancer types (bladder, breast, colon, rectum, lung, cervix, and head and neck) would increase by 6–8% even with a delay of only 4-week in the curative treatment. Also, several studies showed an increase by up to 5-fold of the incidence of cardiovascular diseases complications as out-of-hospital cardiac arrest after the launch of emergency response measures against COVID-19 (20–22). Also, after the implementation of the lockdown, hospital admissions among diabetic patients decreased (23, 24) with a 92% increase in the average number of monthly deaths highlighted by Hernandez-Vasquez et al. (24) and a significantly higher plasma glucose and HbA1c levels than during pre-pandemic period (23).

Therefore, in the light of these considerations, the aim of our study was to investigate the impact of the COVID-19 pandemic on the healthcare and the disease management of patients affected by NCDs, by exploring, specifically, the obstacles encountered in the access to healthcare services during the latest phase of the pandemic.

## 2. Materials and methods

### 2.1. Study design and population

The survey took place from October 2021 to February 2022 in a Teaching Hospital of Southern Italy. To be included in the study, subjects had to be affected by at least one NCD, such as diabetes, hypertension, respiratory and heart diseases, renal and liver chronic conditions, immunodeficiency disorders due to cancer, or being kidney or liver transplant recipients. Patients affected by conditions managed by specific healthcare service areas, such as mental disorders, sexual, reproductive, maternal, child and adolescent health, were excluded.

All eligible subjects attending the anti-SARS-CoV2 vaccination clinic in the Teaching Hospital were asked to participate in our study. The proposal to participate in the study was made in the waiting room while the patients waited their turn. Prior to face-to-face interviews, the research team presented the aims of the study, emphasized that participation was voluntary and the anonymity of the responses and acquired written consent.

The sample size was estimated using the following equation: *n* = (*z*^2^ × *p* × *q*)/*D*^2^ (25) with the following assumptions: the probability of obstacles in healthcare accesses in NCDs patients was estimated to be equal to 25% (p), a value of 0.5 was chosen as the acceptable limit of precision (D) with a confidence interval of 99%(z). The value obtained was increased by 10% to take into account the non-response rate. Based on these assumptions the final sample size became 550 NCDs patients.

### 2.2. Survey instrument

Data were collected through a questionnaire administered to all subjects who gave their consent to participate in the study. The questionnaire consisted of three sections: in the first one, items on sociodemographic characteristics and lifestyle habits were included; in the second section, the health status was investigated and, to quantify the individual burden of disease, the age adjusted Charlson co-morbidity index was calculated for each subject. The perceived health status at the time of the interview was measured on a ten-point Likert scale for response, ranging from 0 (very poor) to 10 (excellent). In the third section the health status and the utilization of healthcare services during the six months prior to the subject’s enrollment in the study were investigated. In particular, information was collected about general practitioner (GP) and/or specialist consultations; hospital admissions; eventual obstacles in the access to healthcare services, including canceled/postponed medical consultations, follow-up visits, scheduled surgery and preventive activities. Finally, reasons for difficult access to healthcare services were investigated.

The study protocol was ratified by the Approval from the Regional Ethics Committee (ID N. 183).

### 2.3. Statistical analysis

Descriptive analyses were performed to illustrate demographic characteristics of participants and to determine the prevalence of the experienced obstacles in the access to healthcare services. Moreover, univariate analysis, by the appropriate test (*t*-test; chi-square test), and a multivariate stepwise logistic regression model were performed to determine the independent association of several characteristics with the experienced obstacles in the access to healthcare services, during the previous 6 months. The following explanatory variables were included in the model: gender (male = 0; female = 1), age (continuous), additional person in the household (none = 0; ≥1 = 1), marital status (married/cohabitant = 0; other = 1), education level (primary/secondary school = 0; high school = 1; university degree = 2), cardiovascular disease (no = 0; yes = 1), diabetes mellitus (no = 0; yes = 1), gastrointestinal diseases (no = 0; yes = 1), immunodeficiency disorders (no = 0; yes = 1), kidney disease (no = 0; yes = 1), neurological disease (no = 0; yes = 1), cancer (no = 0; yes = 1), respiratory diseases (no = 0; yes = 1), perceived health status (poor/fair/good = 0; very good/excellent = 1), number of chronic diseases (<3 = 0; ≥3 = 1), consultation to GP (none = 0; at least one = 1) and to the specialists (none = 0; at least one = 1) in the previous six months. Adjusted Odds Ratio (OR) and 95% Confidence Intervals (95% CI) were calculated. A *p* value ≤0.05 was considered statistically significant.

STATA version 16 statistical software package (2016, Stata Corp, LP, College Station, Tx, United States) was used to conduct all data analyses.

## 3. Results

A total of 553 subjects completed the questionnaire, with an 87% response rate. Selected characteristics of the sample are reported in Table 1. The mean age was 66.8 years (± 12.1 SD); 45.6% (95% CI 41.4–49.8) of respondents declared to suffer from three or more NCDs and the most prevalent were cardiovascular illness, including hypertension and heart diseases (77.4, 95% CI 73.7–80.8); cancer (28.2, 95% CI 24.5–32.2) and diabetes (25.5, 95% CI 21.9–29.3). Primary and secondary immunodeficiency disorders due to transplantation, asplenia and immunosuppressive medications were reported by 18.6% (95% CI 15.3–21.9) of respondents. Moreover, when patients were asked to indicate in a 10-point Likert-type scale their perceived health status, 39.2% (95% CI 34.4–42.7) evaluated their health in general as very good or excellent.

**Table 1:** Demographics and baseline characteristics of the study population and their association with prevalence of the experienced obstacles in the access to healthcare services during the latest phase of the COVID-19 pandemic.

Characteristic	Total (553)		Experienced obstacles in the access to healthcare services	
	N, prevalence (95% CI)		*No*	*Yes*
*Socio-demographic profile*				
**Gender**				
Male	273, 49.4 (45.1–53.6)		171 (62.6)	102 (37.3)
Female	280, 50.6 (46.4–54.9)		164 (58.6)	116 (41.4)
			χ2 = 0.956, 1df, *p* = 0.328	
**Age, years Mean ± SD**	66.8 ± 12.1		67.03 ± 12.3	66.4 ± 11.7
			*t*-test = − 0.648, 551df, *p* = 0.516	
20–55	87, 15.7 (12.8–19)		52 (59.8)	35 (40.2)
56–65	141, 25.5 (21.9–29.3)		87 (61.7)	54 (38.3)
66–75	199, 36 (32–40.1)		114 (57.3)	85 (42.7)
>75	126, 22.8 (19.4–26.5)		82 (65.1)	44 (34.9)
			χ^2^ = 2.07, 3df, *p* = 0.558	
**Marital status (546)**				
Married	426, 78 (74.3–81.4)		256 (60.1)	170 (39.9)
Other	120, 22 (18.6–25.7)		76 (63.3)	44 (36.7)
			χ^2^ = 0.412, 1df, *p* = 0.521	
**Additional persons in the household (545)**				
None	86, 15.8 (12.8–19.1)		59 (68.6)	27 (31.4)
≥1	459, 84.2 (80.9–87.2)		273 (59.4)	186 (40.6)
			χ^2^ = 2.534, 1df, *p* = 0.111	
**Education level (543)**				
Primary and Secondary school	229, 42.2 (38–46.5)		135 (59)	94 (41)
High school	213, 39.2 (35.1–43.5)		130 (61)	83 (39)
University graduate	101, 18.6 (15.4–22.1)		66 (65.4)	35 (34.6)
			χ^2^ = 1.205, 2df, *p* = 0.547	
**Working activity (547)**				
Retired	415, 75.9 (72.1–79.4)		247 (59.5)	168 (40.5)
Employed	132, 24.1 (20.1–27.9)		85 (64.4)	47 (35.6)
			χ^2^ = 0.998, 1df, *p* = 0.318	
*Behavioral risk factors and medical conditions*				
**Smoking habits (552)**				
Never/past smoker	505, 91.5 (88.8–93.7)		302 (59.8)	203 (40.2)
Current smoker	47, 8.5 (6.3–11.2)		33 (70.2)	14(29.8)
			χ^2^ = 1.953, 1df, *p* = 0.162	
**Alcohol use (543)**				
No	312, 57.5 (53.2–61.7)		194 (62.2)	118 (37.8)
Yes	231, 42.5 (38.3–46.8)		135 (58.4)	96 (41.6)
			χ^2^ = 0.776, 1df, *p* = 0.378	
**Cardiovascular diseases**				
No	125, 22.6 (19.2–26.3)		76 (60.8)	49 (39.2)
Yes	428, 77.4 (73.7–80.8)		259 (60.5)	169 (39.5)
			χ2 = 0.003, 1df, *p* = 0.954	
**Diabetes**				
No	412, 74.5 (70.7–78.1)		260 (63.1)	152 (36.9)
Yes	141, 25.5 (21.9–29.3)		75 (53.2)	66 (46.8)
			χ^2^ = 4.324, 1df, *p* = **0.038**	
**Gastrointestinal diseases**				
No	489, 88.4 (85.5–91)		303 (61.9)	186 (38.1)
Yes	64, 11.6 (9–14.5)		32 (50)	32 (50)
			χ2 = 3.391, 1df, *p* = 0.066	
**Immunodeficiency disorders**				
No	451, 81.6 (78.1–84.7)		283 (62.7)	168 (37.3)
Yes	102, 18.6 (15.3–21.9)		52 (51)	50 (49)
			χ2 = 4.824, 1df, *p* = **0.028**	
**Kidney diseases**				
No	470, 85 (81.7–87.9)		281 (59.8)	189 (40.2)
Yes	83, 15 (12.1–18.3)		54 (65.1)	29 (34.9)
			χ2 = 0.821, 1df, *p* = 0.365	
**Neurological diseases**				
No	515, 93.1 (90.7–95.1)		316 (61.4)	199 (38.6)
Yes	38, 6.9 (4.9–9.3)		19 (50)	19 (50)
			χ2 = 1.912, 1df, *p* = 0.167	
**Cancer**				
No	397, 71.8 (67.8–75.5)		235 (59.2)	162 (40.8)
Yes	156, 28.2 (24.5–32.2)		100 (64.1)	56 (35.9)
			χ2 = 1.129, 1df, *p* = 0.288	
**Respiratory diseases**				
No	482, 87.2 (84.1–89.8)		297 (61.6)	185 (38.4)
Yes	71, 12.8 (10.2–15.9)		38 (53.5)	33 (46.5)
			χ2 = 1.699, 1df, *p* = 0.192	
**Age-adjusted Charlson Comorbidity Index (CCIa)**				
Mean ± SD	5.15 ± 1.98		5.1 ± 2.04	5.2 ± 1.91
			t-test = − 0.782, 551df, *p* = 0.434	
**Number of NCDs**				
< 3	301, 54.4 (50.2–58.6)		194 (64.5)	107 (35.5)
≥ 3	252, 45.6 (41.4–49.8)		141 (55.9)	111 (44.1)
			χ^2^ = 4.149, 1df, *p* = **0.042**	
**Perceived health status**				
Poor/Fair/Good (≤7 points)	330, 60.8 (55.5–63.8)		205(62.1)	125(37.9)
Very good/Excellent (≥8 points)	213, 39.2 (34.4–42.7)		126(59.2)	87(40.8)
			χ2 = 0.478, 1df, *p* = 0.489	

SD: standard deviation.

The number of participants responding to the questions is indicated in brackets.

Utilization of healthcare services was referred to the six months prior to the enrollment in the study.

The respondents’ pattern of utilization of healthcare services during the latest phase of the COVID-19 pandemic (Table 2) showed that, in the previous six months before enrollment in the study, almost 90% of the subjects had consulted GP at least once, and the mean reasons were medical prescriptions (77, 95% CI 73–80.6) and routine checks for NCDs (22%), while preventive interventions were required by only 1% of the subjects. Among the 85.5% (95% CI 82.3–88.4) of respondents that reported at least one specialist visit, routine checks for NCDs were the main declared reason (95.4, 95% CI 93–97.1). One third of respondents (33.1, 95% CI 29.2–37.2) were hospitalized, mainly for surgery (45.9, 95% CI 38.5–53.4) or for the occurrence of new symptoms (29.5, 95% CI 23–36.7). Slightly less than half (42.3, 95% CI 32.1–54.1) of the performed surgical interventions were severe/urgent, because they were carried out on cancer patients (54.5, 95% CI 38.1–72.1) or in patients in critical conditions (22.8, 95% CI 10.1–39.2) or because they were transplants (22.7, 95% CI 10.1–39.2) (data not shown).

**Table 2:** Characteristics of the utilization of healthcare services during the latest phase of the COVID-19 pandemic by patients with NCDs.

		Total (553) N, prevalence (95% CI)	
**GP consultation**			
None		62, 11.2 (8.7–14.1)	
Phone contact		110, 19.9 (16.6–23.5)	
At least one access		381, 68.9 (64.9–72.7)	
	*Main reasons for GP consultation (phone and/or access)*		
	Demand for a medical prescription	378, 77 (73–8.6)	
	Routine checks for NCDs	106, 22 (18–25.5)	
	Preventive measures (eg. vaccinations, blood pressure check in non-hypertensives)	7, 1 (0.6–2.9)	
**Specialist accesses**			
None		80, 14.5 (11.6–17.7)	
At least one		473, 85.5 (82.3–88.4)	
	*Main reasons for accesses*		
	Routine checks for NCDs	451, 95.4 (93–97.1)	
	Diagnosis and treatment of new or more complex diseases	20, 4.2 (2.6–6.5)	
	Do not remember	2, 0.4 (0.05–1.5)	
**Hospital admission**			
No		370, 66.9 (62.8–70.8)	
Yes		183, 33.1 (29.2–37.2)	
	*Reasons for hospital admission*		
	Surgery	84, 45.9 (38.5–53.4)	
	Diagnosis and treatment of new disease	54, 29.5 (23–36.7)	
	Scheduled check for to pre-existing NCDs	23, 12.6 (8.1–18.3)	
	Exacerbation of pre-existing NCDs	22, 12 (7.7–17.6)	
**Experienced obstacles in the access to healthcare services**			
No		335, 60.6 (56.4–64.7)	
Yes		218, 39.4 (35.3–43.6)	
	*Type of cancelled/postponed healthcare service (226)* ^a^		
	Routine check for NCDs	137, 60.6 (53.9–67)	
	Follow-up of more complex diseases (eg. cancer, transplantation)	39, 17.3 (12.6–22.8)	
	Scheduled surgery	26, 11.5 (7.7–16.4)	
	Preventive screening	10, 4.4 (2.1–8)	
	Evaluation of new symptoms	9, 4 (1.8–7.4)	
	Do not remember	5, 2.2 (0.7–5.1)	
	*Reasons for cancelled/postponed healthcare service*		
	Temporary closure of elective healthcare activities	123, 56.4 (49.6–63.1)	
	Fear of being infected	59, 27 (21.3–33.5)	
	Positive COVID-19 test of patients/health professionals	13, 6 (3.2–10)	
	Inconvenience in reaching the service	5, 2.3 (0.7–5.3)	
	Do not remember	18, 8.3 (5–12.7)	

Utilization of healthcare services was referred to the six months prior to enrollment in the study.

a The total of canceled/postponed healthcare services exceeded the total of subjects because one or more services could have been deferred.

**Figure 1:**
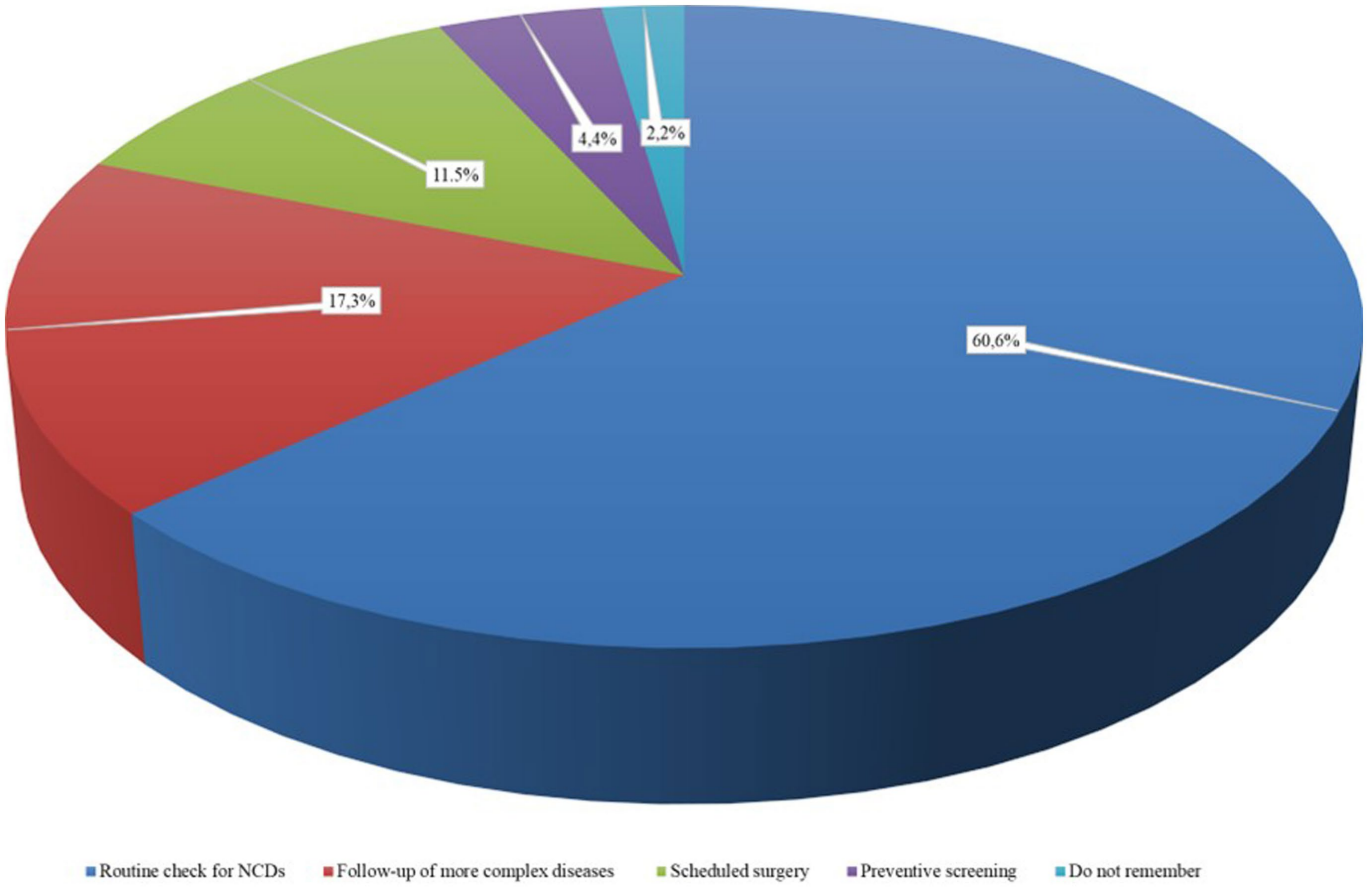
Types of canceled/posponed healthcare services during the latest phase of the COVID-19 pandemic.

**Figure 2:**
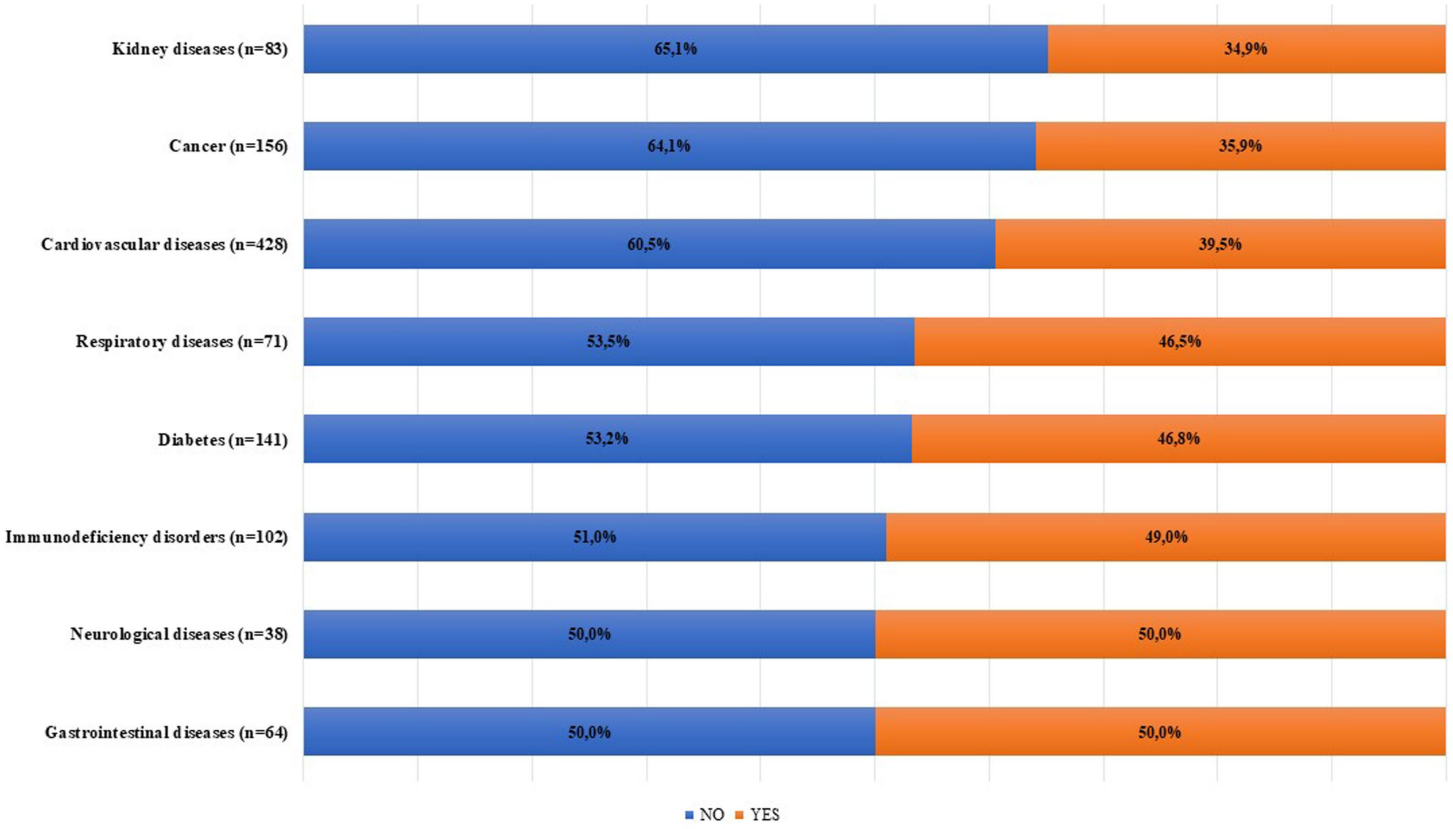
Experienced obstacles in the access to healthcare services according to NCDs during the latest phase of the COVID-19 pandemic.

Overall, 39.4% (95% CI 35.3–43.6) of the patients experienced obstacles in the access to healthcare services in the six months prior to the enrollment in the study. The most frequent canceled/postponed healthcare services were the visits for routine checks for NCDs (60.6, 95% CI 53.9–67), followed by control visits of more complex diseases as cancer or transplantation (17.3, 95% CI 12.6–22.8) and scheduled surgery (11.5, 95% CI 7.7–16.4) (Figure 1) and, of these, more than a quarter (26.5, 95% CI 11.6–47.8) were not performed (data not shown). Preventive interventions and the evaluation of the occurrence of new symptoms accounted for lower than 10% of the canceled healthcare services. The temporary closure of elective healthcare activities was the most common reason, reported by 56.4% (95% CI 49.6–63.1) of the patients that had experienced canceled or postponed healthcare services. In the Figure 2 were reported the percentages of patients who experienced obstacles in the access to healthcare services according to chronic disease suffered (Figure 2).

Univariate analysis (Table 1) highlighted that patients who experienced canceled/postponed healthcare services in the latest phase of the COVID-19 pandemic were significantly more likely to suffer from 3 or more NCDs (χ^2^ = 4.149, *p* = 0.042), to be diabetics (χ^2^ = 4.324, *p* = 0.038), to have immunodeficiency disorders (χ^2^ = 4.824, *p* = 0.028) and to have consulted GP at least once (χ^2^ = 8.292, *p* = 0.004; data not shown), while no significant differences were found according to the socio-demographic profile or other NCDs.

When the multivariate analysis was performed, the results did not substantially change, with the exception of patients not living alone and those affected by respiratory diseases who had significantly higher odds of experiencing canceled/postponed healthcare services (Table 3).

**Table 3:** Multiple stepwise logistic regression analysis results.

Variable	OR	SE	95% CI	*p* value
**Outcome: Experienced obstacles in the access to healthcare services** *Log-likelihood = −334.04, Chi-square = 37.28, p = 0.0001, N of obs = 528*				
**Additional persons in the household**				
No	1.00*			
Yes	1.78	0.48	1.05–3.01	***0.033***
**Gender**				
Male	1.00*			
Female	1.48	0.29	0.99–2.20	0.052
**GP consultation**				
None	1.00*			
At least one	2.53	0.84	1.32–4.86	***0.005***
**Specialist accesses**				
None	1.00*			
At least one	0.64	0.17	0.38–1.09	0.105
**Perceived health status**				
Fair/Poor/Good (≤7 points)	1.00*			
Very good/Excellent (≥8 points)	1.22	0.24	0.83–1.80	0.305
**Immunodeficiency disorders**				
No	1.00*			
Yes	1.87	0.48	1.13–3.07	***0.014***
**Gastrointestinal diseases**				
No	1.00*			
Yes	1.97	0.56	1.12–3.45	***0.019***
**Diabetes**				
No	1.00*			
Yes	1.58	0.34	1.04–2.40	***0.032***
**Respiratory diseases**				
No	1.00*			
Yes	1.76	0.49	1.01–3.05	***0.044***
**Kidney diseases**				
No	1.00*			
Yes	0.67	0.19	0.39–1.16	*0.153*
**Neurological diseases**				
No	1.00*			
Yes	1.40	0.53	0.67–2.96	0.371

SD: standard deviation; GP: general practitioner.

*Reference category.

Utilization of healthcare services was referred to the six months prior to the enrollment in the study.

The following variables were removed from the model by backward elimination procedure: age, marital status, education level, cardiovascular diseases, oncological diseases, number of NCDs.

## 4. Discussion

It is well known that severity and mortality associated to SARS-CoV2 infections increase dramatically with older age (>65 years), and in subjects suffering with pre-existing NCDs (26, 27). Many people, especially those in the above mentioned high-risks groups had experienced obstacles in the access to healthcare services to obtain treatment for ongoing conditions during the COVD-19 pandemic. Although this result is reasonable in the early phase of the pandemic, because of the prioritization of healthcare services against SARS-CoV2 infections, it is less acceptable in the latest phase of the pandemic. Indeed, the most remarkable result from this study revealed the persistent reduction of the elective healthcare activities, with almost 40% of patients with NCDs having experienced canceled/postponed healthcare services during this period.

To the best of our knowledge this investigation is the first offering an overview of how frail patients continued to experience obstacles in the access to healthcare services even when infection control measures, as mandatory use of protective masks, physical distancing, quarantine measures, had become less stringent and the effectiveness of the vaccination campaigns in the prevention of the most severe clinical outcomes had become clearly evident.

In this study, two main obstacles to access to healthcare services during the latest phase of the pandemic have emerged: the persistence of temporary closure of elective healthcare activities, and a specific patients’ choice due to the fear of being infected. Regarding the first aspect, as the more recent WHO interim report (28) has suggested, worldwide healthcare systems have not yet overcome the acute phase of the pandemic. Despite lower direct mortality and hospitalization than in the initial phase of the pandemic, the SARS-CoV2 infection continues to disrupt essential healthcare services in almost every country across the world (29). It is reasonable to hypothesize that the lack of resources, in terms of availability of health workers, drugs, vaccines, except for the anti-SARS-CoV2 ones, but also of facility infrastructure, space capabilities and diagnostic equipment could, at least in part, explain the persistent dearth of elective healthcare services.

The Italian National Healthcare System (NHS), as well as that of several other Organization for Economic Co-operation and Development (OECD) countries, has implemented the waiting list model, based on prioritization criteria according to clinical conditions and professional judgment, to manage the population healthcare requests (30). A non-secondary problem in the current healthcare landscape is represented by the need to make up for the delay accumulated in the management of waiting lists during the complete lockdown phase. Currently, indeed, the healthcare services requests add up to those planned but not carried out during the pandemic emergency phase and the subjects who most suffer to wait longer are those with worse health status, as recently reported by Brito Fernandes et al. (31). Therefore, obstacles experienced in the latest phase of the pandemic could be the consequences of pre-existing healthcare systems problems that have been exacerbated by the pandemic. Indeed, across all OECD countries in 2019, spending on prevention accounted for only 2.7% of the total health spending; healthcare systems were understaffed with an average of 12.4 of doctors and nurses per 1,000 people, and they suffered from underinvestment with insufficient physical infrastructure and occupancy rate over 90% for acute care beds or countries with less than half the mean expected number of adult intensive care beds (32, 33).

Similarly, to previous studies (34–36), almost 27% of NCDs patients avoided/delayed healthcare services due to the fear of being infected with SARS-CoV2, and this attitude was more frequent in patients with multiple medical conditions compared to those with a lower comorbidity burden (37). This is of particular concern, as timely interventions for these chronic conditions could reduce morbidity and mortality while, conversely, delay in healthcare access may adversely affect evolution of chronic conditions with potentially lasting consequences even after the pandemic.

During the earlier phases of the pandemic, self-management behaviors were encouraged among NCDs patients (38–40). Indeed, the self-management of interventions such as physical activity, diet, pharmacological treatment, monitoring of symptoms, is recognized as a promising measure, capable of improving the ability of NCDs patients in the control of their chronic disease, and resulting in the saving of healthcare resources and the improvement of clinical outcomes, however, despite a vast literature on this topic, there is a paucity of high-quality evidence showing improved outcomes for NCDs patients as a result of the implementation of self-management behaviors (40–42). It is plausible to hypothesize that NCDs patients continued to strictly adhere to self-management behaviors also in the latest phase of the pandemic, so delaying/avoiding healthcare services access. Indeed, as highlighted by the results of the study, diabetic patients, that represent the more routinely trained category in the self-management behaviors (43) and that during the pandemic have substantially increased self-management practices (38, 39, 44), had significantly higher odds of experiencing canceled/postponed healthcare services in respect to non-diabetics patients.

The finding that patients not living alone were more likely to experience canceled/postponed healthcare services is in line with previous studies, which have demonstrated that loneliness is an independent determinant of healthcare services utilization regardless of the subject’s health status (45), and that the physician-patient relationship could be a way to get not only medical care, but social support as well (46). According to Arreskov et al. (47), the role of the GP remains unique in terms of opportunities to establish a stable and close doctor-patient relationship and, above all, to ensure an individualized approach, especially in subjects with chronic and complex health problems. Therefore, it is not surprising our result that cancelation/postposition of the access to healthcare services were carried out more significantly by respondents which have consulted the GP at least one time in the study period.

It is essential to read the findings from this study considering the following possible limitations. First of all, the data were self-reported by patients and, due to the potential recall bias, it could represent a problem in terms of overestimates or underestimates of experienced obstacles in the access to healthcare services. Even though the period in question was limited to the previous 6 months only. Second, among the information collected, it was not asked whether the specialist visits were paid or carried out by public specialists, but this limit does not affect the results of the study which did not have among the objectives of evaluating the use, by patients, of paid visits to obtain healthcare services more quickly. Third, results must be interpreted under the cross-sectional point of view, taking into account its limitations in terms of capability to detect cause-effect relationship. Finally, our study involved only one Italian region, which might not represent all NCDs patients in Italy. However, we are confident the results of the study may be generalized at least to the Southern Italy regions.

## 5. Conclusion

Despite its limitations, this study provides important insights into how the COVID-19 pandemic continues to impact NCDs patients even in its latest and less aggressive phase. If patients with NCDs continue to be hindered in accessing health care services to manage chronic conditions, receive routine immunizations, or diagnose new conditions early, the consequences will be irreparable for the most frail patients (36). It therefore appears essential to provide chronic patients with correct information on the real risks associated with SARS-CoV2 infection with respect to those of non-treatment of the chronic pathologies presented or the onset of new symptoms. Finally, these results appear to be fundamental for guiding the choices of providers in order to concentrate organizational efforts to recover and reschedule missed appointments, where applicable, of the most fragile patients by virtue of age and chronic conditions.

## Data availability statement

The raw data supporting the conclusions of this article will be made available by the authors, without undue reservation.

## Ethics statement

The studies involving humans were approved by Regional Ethics Committee (Calabria Region). The studies were conducted in accordance with the local legislation and institutional requirements. The participants provided their written informed consent to participate in this study.

## Author contributions

CS: Conceptualization, Investigation, Supervision, Data curation, Formal analysis, Writing – original draft. RP: Data curation, Formal analysis, Investigation, Writing – original draft. MM: Data curation, Investigation, Writing – original draft, Software. CP: Investigation, Conceptualization, Methodology, Supervision, Validation, Writing – review & editing.
